# Daytime Sleepiness: Associations with Alcohol Use and Sleep Duration in Americans

**DOI:** 10.1155/2014/959152

**Published:** 2014-01-29

**Authors:** Subhajit Chakravorty, Nicholas Jackson, Ninad Chaudhary, Philip J. Kozak, Michael L. Perlis, Holly R. Shue, Michael A. Grandner

**Affiliations:** ^1^MIRECC VISN-4, Philadelphia Veterans Affairs Medical Center, University & Woodland Avenues, Philadelphia, PA 19104, USA; ^2^Perelman School of Medicine, University of Pennsylvania, Philadelphia, PA 19104, USA; ^3^University of Southern California, Los Angeles, CA 90033, USA; ^4^West Chester University of Pennsylvania, West Chester, PA 19383, USA; ^5^School of Veterinary Medicine, University of Pennsylvania, Philadelphia, PA 19104, USA; ^6^Children's Hospital of Philadelphia, Philadelphia, PA 19104, USA

## Abstract

The aim of the current analysis was to investigate the relationship of daytime sleepiness with alcohol consumption and sleep duration using a population sample of adult Americans. Data was analyzed from adult respondents of the National Health and Nutritional Examination Survey (NHANES) 2007-2008 (*N* = 2919) using self-reported variables for sleepiness, sleep duration, and alcohol consumption (quantity and frequency of alcohol use). A heavy drinking episode was defined as the consumption of ≥5 standard alcoholic beverages in a day. Logistic regression models adjusted for sociodemographic variables and insomnia covariates were used to evaluate the relationship between daytime sleepiness and an interaction of alcohol consumption variables with sleep duration. The results showed that daytime sleepiness was reported by 15.07% of the subjects. In univariate analyses adjusted for covariates, an increased probability of daytime sleepiness was predicted by decreased log drinks per day [OR = 0.74 (95% CI, 0.58–0.95)], a decreased log drinking frequency [0.90 (95% CI, 0.83–0.98)], and lower sleep duration [OR = 0.75 (95% CI, 0.67–0.84)]. An interaction between decreased sleep duration and an increased log heavy drinking frequency predicted increased daytime sleepiness (*P* = 0.004). Thus, the effect of sleep duration should be considered when evaluating the relationship between daytime sleepiness and heavy drinking.

## 1. Introduction

Daytime sleepiness is highly prevalent. 19.5% of Americans suffer from moderate sleepiness and 11% suffer from severe sleepiness [1]. These prevalence rates are of concern given that daytime sleepiness affects nearly every aspect of human functioning and is a substantial risk factor for accidents and injuries [2]. The combination of daytime sleepiness and at-risk vocations vulnerable to such negative effects magnifies the risk for adverse outcomes of accidents, for example, sleepiness in truck drivers, airline pilots, medical personnel, mass transit operators, and so forth [3]. The perils associated with these conditions underscore the need to identify factors that may serve to aggravate sleepiness, or may serve as vulnerability for sleepiness. One such condition may be the use of or the abuse of alcohol.

To date, some studies have been undertaken to evaluate the relationship between daytime sleepiness and alcohol consumption. The outcomes from these studies were inconsistent. Acute drinking episodes were found to be associated with complaints of sleepiness (as a component of hangover symptoms) [4] and an acute impairment in objective measures of flying the following day (after an acute drinking episode) [5]. In contrast, increased alcohol consumption (>7 drinks per week) has been shown to be associated with a decreased likelihood of excessive daytime sleepiness in the elderly [6]. These studies differed in several ways, including the following: the duration of alcohol use (i.e., acute versus chronic use), the age of the cohort studied, and the sleep duration immediately prior to the study (or the typical total sleep duration).

The last of these issues is the role of sleep duration, which is particularly important as an increased consumption of alcohol has been independently associated with short sleep duration in some prior epidemiological studies [7–9]. Sleep duration has been investigated as a moderator in this relationship between alcohol consumption and sleepiness in a few laboratory-based studies. Some of these studies involved paradigms with partial sleep restriction and some with sleep extension (longer than typical ad lib sleep). In the *partial sleep deprivation studies*, Rupp and colleagues found that nocturnal alcohol consumption prior to bedtime and in conjunction with a partial sleep restriction increased the sleepiness at night (as compared to those who did not consume alcohol) [10]. Roehrs and colleagues found that despite an increase in the sleep latency on the Multiple Sleep Latency Test (MSLT) with partial sleep deprivation and moderate nocturnal alcohol consumption, no interaction between alcohol consumption and sleep deprivation was seen [11]. Similarly, a study by Horne and colleagues failed to show a difference in daytime sleepiness in the sleep-deprived conditions, with or without afternoon alcohol consumption, by using a different paradigm [12]. In the *sleep extension studies*, Lumley and colleagues found no differences in objective sleepiness on MSLT in subjects with an 11-hour total time in bed and morning alcohol consumption (as compared to normal sleep or partial sleep deprivation conditions) [13]. In another study, Roehrs and colleagues found that sleep extension with a 10-hour time in bed and morning alcohol consumption showed a decreased objectively measured sleepiness with a MSLT test [14].

As can be seen from the above summary, the data to date are mixed. If trends are evident it appears that heavy alcohol consumption is associated with shorter sleep durations, and objective daytime sleepiness may be associated with acute alcohol use/alcohol abuse in association with sleep deprivation. These findings must be considered tentative, as there are only a few studies with mixed results, and the alcohol measures are not well operationalized. With respect to the alcohol measures, the variables used rarely allow for a comprehensive assessment that takes into account dose (amount of alcohol per occasion), use frequency (number of occasions using alcohol per day or week), and use in the hazardous range (the presence of heavy drinking, and the frequency of heavy drinking as defined as the use of ≥5 drinks per session) [15, 16].

Accordingly, using a nationally representative sample, we explored the interactions between self-reported alcohol consumption and sleep duration variables and their association to daytime sleepiness while controlling for covariates and symptoms related to several intrinsic sleep disorders.

## 2. Methods

### 2.1. Design and Setting

This investigation utilized the 2007-2008 National Health and Nutrition Examination Survey (NHANES). This annual survey, conducted by the Centers for Disease Control and Prevention, assesses the demographic, health, and nutritional characteristics in the US population through in-person interviews, physical examinations, and laboratory tests. The unweighted response rate for the overall sample was 78.4%. In order to compensate for under-representation, African Americans, Hispanics, and adults over 60 were over-sampled [17].

### 2.2. Sample Size

Out of the initial participants (*N* = 10,149), we excluded children and adolescents <18 years of age (*N* = 3921), those with a lifetime history of drug use (*N* = 1896), those without response to outcome variables (*N* = 21), and those missing data on predictor variables (*N* = 1079), and covariates (*N* = 313). The final sample consisted of 2919 subjects. Drug use history was assessed for any lifetime use of marijuana, cocaine, methamphetamine, or illicit opiates individually; the response was coded dichotomously as “yes”/“no.”

### 2.3. Measures

#### 2.3.1. Sleep

(a) Daytime sleepiness (*DS*) was assessed with the question, “In the past month, how often did you feel excessively or overly sleepy during the day?” (b) Sleep Duration (*SD*) was investigated with the question, “How much sleep do you usually get at night on weekdays or workdays?” This question was similar to that used in prior studies [18, 19]. (c) Insomnia symptoms: (i) difficulty falling asleep (*DFA*) was assessed using the question “In the past month, how often did you have trouble falling asleep?” (ii) difficulty maintaining sleep (*AWAK*) was assessed with the question “In the past month, how often did you wake up during the night and had trouble getting back to sleep?” (iii) Nonrestorative sleep (*NRS*) was evaluated with the question,“In the past month, how often did you feel unrested during the day, no matter how many hours of sleep you have had?” The responses to the DS, SL, AWAK, and SQ variables were presented with the following severity options: “0” (never), “1” (rarely: 1 time/month), “2” (sometimes: 2–4 times/month), “3” (often: 5–15 times/month), and “4” (almost always: 16–30 times/month). The response to the SD variable was recorded as a number, rounded to the nearest decimal point and was assessed as a continuous variable.

#### 2.3.2. Alcohol-Related Variables

The quantity of alcohol in a drink was evaluated in terms of a standard alcoholic drink [20]. The alcohol consumption variables used in this investigation were in line with those used in prior studies, and included the following [16, 21]: (a) drinks/day: this variable was assessed with “In the past 12 months, on those days that you drank alcoholic beverages, on an average, how many drinks did you have?” (b) drinking frequency was investigated using the question “In the past 12 months, how often did you drink any type of alcoholic beverage?” (c) heavy drinking status was assessed using the question “In the past 12 months, on how many days did you have 5 or more drinks of any alcoholic beverage?” (d) heavy drinking frequency was evaluated using the question, “In the past 12 months, on how many days did you have 5 or more drinks of any alcoholic beverage?” Drinks/day, drinking frequency and binge-drinking frequency were recorded continuously as number of days. Heavy drinking status was recorded dichotomously, as “present” or “absent.”

#### 2.3.3. Covariates

The variables included in these analyses included, age, gender, race/ethnicity (White, Black/African American, Hispanic, and other), marital status, education, income, body mass index (BMI; objectively measured), depression (over past two weeks), anxiety (days anxious in past month), access to health insurance, physical health, mental health, exercise, and smoking (smoking days in past month). All the above-mentioned questions were assessed as part of the NHANES interview with the responses being self-reported by the subjects.

### 2.4. Statistical Analysis

Two-year full sample weights were used to adjust for unequal probability of being selected among noncoverage or nonresponse population, as recommended [17]. Daytime sleepiness was assessed with the question, “In the past month, how often did you feel excessively or overly sleepy during the day?” Daytime sleepiness was assessed as a dichotomous variable (“presence” or “absence” of daytime sleepiness) based on the distribution of the response. The sleep duration was assessed as a continuous variable. Alcohol consumption was assessed using four variables, including, drinks/day, drinking frequency, heavy drinking status, and heavy drinking frequency. Heavy drinking status was assessed as a dichotomous variable (“presence” or “absence” of heavy drinking). The remaining 3 alcohol variables were assessed as continuous variables, drinks/day, drinking frequency, and heavy drinking frequency. Log_*n*_ transformation was conducted for the variables including, drinks/day, drinking frequency, and heavy drinking frequency because of the skewness in the data, prior to the bivariate and multivariable analyses. Some of the covariates were dichotomized because of the skewed distribution and included insomnia variables (reporting a complaint ≥5 times a month/<5 times a month), depression, and anxiety symptoms (symptoms <15 days/≥15 days over last month). The relationships between daytime sleepiness (dependent variable) and alcohol consumption variables were assessed using multinomial logistic regression analyses. This relationship was assessed using three different models to adjust for covariates. Model 1 assessed the crude relationship between daytime sleepiness and the alcohol consumption variables or sleep duration. Model 2 assessed the relationship in model 1, adjusted for the covariates mentioned above. Model 3 assessed for this relationship in Model 2, further adjusted for insomnia symptoms. Interactive models evaluated for the presence of 2-way interactions, of whether the effects of one alcohol consumption variable depended on levels of a second category, that is, the sleep duration in predicting daytime sleepiness. Analyses were conducted using Stata version 12 (StataCorp LP, Stata Statistical Software: Release 12. College Station, TX).

## 3. Results

### 3.1. Subjects

The average subject in this study which was middle-aged female, college graduate, who identified herself as of Caucasian race, non-Hispanic in ethnicity, married, was overweight and had health insurance, Table 1.

### 3.2. Sleep-Related Characteristics

Daytime sleepiness was reported by 15.07% of the subjects. The mean (SD) sleep duration was 6.91 (SD = 1.36) hours. Amongst them, 56.72% had sleep duration within the normal range (7-8 hours a night), 36.12% had short sleep duration (≤6 hours a night), and 7.16% had long sleep duration (≥8 hours a night), with nonrestorative sleep being the commonest insomnia symptom (22.32%), Table 1.

### 3.3. Alcohol Consumption

The average subject reported an alcohol consumption in the moderate range with a mean (SD) alcohol consumption of 1.25 (SD = 2.28) drinks per day within the last 12 months. Amongst those drinking alcohol in the past 12 months, 9.77% of the respondents reported heavy drinking (≥5 drinks a day), and with a heavy drinking frequency of 4.23 (SD = 27.18) days of over the last 12 months, Table 1.

### 3.4. The Relationship of Daytime Sleepiness with Alcohol Consumption and Sleep Duration

#### 3.4.1. Alcohol Consumption

Subjects with daytime sleepiness reported lower alcohol consumption as compared to those without daytime sleepiness (0.99 ± 1.65 drinks and 1.29 ± 2.38 drinks resp., *P* = 0.002). In analyses adjusted for sociodemographic variables and insomnia covariates, a decreased risk of daytime sleepiness was predicted by log drinks per day, that is, each percent increase in the number of alcoholic drinks per day [OR = 0.74 (95% CI, 0.58–0.95), *P* = 0.019]. A similar relationship of a decreased risk of daytime sleepiness was seen with increased log drinking frequency, that is, each percent increase in the frequency of drinking and with nonsignificant trends for log binge-drinking frequency predicting a lower risk of daytime sleepiness, Table 2.

#### 3.4.2. Sleep Duration

Those with daytime sleepiness reported a lower sleep duration as compared to those without daytime sleepiness (6.38 ± 1.63 hours and 7.00 ± 1.28 hours, resp., *P* < 0.0001). In analyses adjusted for sociodemographic variables and insomnia covariates, a decreased probability of daytime sleepiness was predicted by higher sleep duration [OR = 0.75 (95% CI, 0.67–0.84), *P* < 0.001], Table 2.

### 3.5. Interactions between Alcohol Consumption and Sleep Duration on Daytime Sleepiness

**Figure 1:**
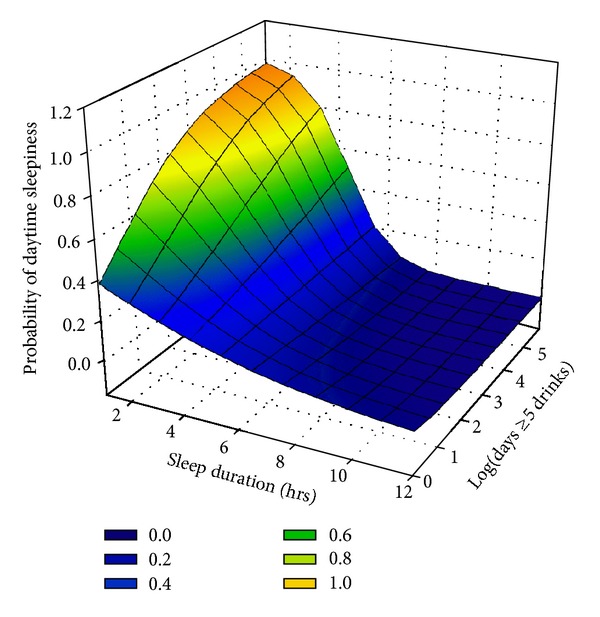
Surface model of the interaction between heavy drinking frequency and sleep duration on daytime sleepiness.

In models adjusted for covariates, an interaction between a decreased sleep duration and an increased log frequency of binge-drinking predicted increased daytime sleepiness (*P* = 0.004), such that with each percent increase in the binge-drinking frequency and a decrease in the sleep duration in hours, there was an increased probability of reporting daytime sleepiness; see Table 3, and Figure 1. No significant interactions between other alcohol consumption variables and sleep duration predicted daytime sleepiness.

## 4. Discussion

The association between subjective sleepiness as it relates to alcohol consumption and sleep duration from a population perspective is currently unknown. In this study, we explored this relationship using data from the 2007-2008 NHANES survey using self-reported measures. In univariate analyses, the presence of daytime sleepiness was inversely associated with the drinks per day, drinking frequency, and sleep duration. In the final model adjusted for covariates, an interaction between heavy drinking frequency and sleep duration predicted daytime sleepiness, such that an increased probability of daytime sleepiness was reported with each percent increase in the frequency of heavy drinking and a decrease in the sleep duration (in hours).

Short sleep duration has been linked with heavy alcohol consumption on one hand [7–9] and with daytime drowsiness on the other hand [22, 23]. Heavy alcohol consumption has been linked with next day symptoms of tiredness [24], and with an impaired performance [5]. It is therefore possible that sleepiness is reliably produced with a higher intensity and periodicity of alcohol consumption along with insufficient habitual sleep duration. The sleep duration may be decreased by the heavy alcohol consumption itself, or from insufficiency based on the need or opportunity of functioning in a 24-hour society, and/or the presence of intrinsic sleep disorders like insomnia or obstructive sleep apnea syndrome. In light of the above it is easier to comprehend our findings of an interaction between heavy drinking and a decreased sleep duration predicting sleepiness.

Evaluating this relationship from another perspective, it is possible that after the alcohol is metabolized in the latter half of the night, the sleep is shallow and fragmented sleep as shown previously [24, 25]. This shallow and/or fragmented sleep may lead the subject to a state of subacute sleep deprivation with continued heavy drinking over time, leading to complaints of daytime sleepiness [4]. Our results differ from those of Pack and colleagues [6] as their study did not account for the sleep duration or for any heavy drinking and showed results similar to our bivariate analysis. In addition, our study adjusted for the effect of gender as a covariate, as well as body mass index and insomnia symptoms in the analyses.

Some of the limitations associated with this study include the following: the cross-sectional nature of the study precludes determination of the cause and effect between the variables; the dichotomous nature of sleepiness complaint prevents us from differentiating relationships associated with varying intensities of the daytime sleepiness; the lack of additional data on the drinking pattern across genders (in a calendar format) over the past year (in days, months, or years) or the pattern of drinking on weekdays versus weekends; and the lack of data on the circadian pattern of sleep and sleepiness as well as caffeine and alcohol consumption. Despite its weaknesses, this is one of the first studies at the population level that shows the presence of a complex relationship between alcohol consumption and sleep duration, on daytime sleepiness.

In conclusion, an inverse relationship of the probability of daytime sleepiness with the intensity and the frequency of alcohol consumption was seen in adult respondents from a nationally representative US sample. Once the duration of sleep was factored in, an interaction between the frequency of heavy drinking and sleep duration predicted an increased probability of daytime sleepiness. These results extend the findings from prior laboratory-based studies to a population sample. Future studies will need to further clarify this complex relationship further using more detailed information on alcohol use, sleepiness in the context of circadian phase and the ascending versus the descending limbs of alcohol concentrations as seen in a prior laboratory study [26], and the association with hangover symptoms. In addition, studies are also warranted to tease apart the role of gender differences in this relationship considering the gender related differences in sleepiness and alcohol consumption.

## Figures and Tables

**Table 1:** Baseline demographics.

Variable	Categories	Mean/%	S.D.
Age (years)		53.1	17.4
Gender	Female	57.84%	
Race	White	68.05%	
	Black	10.20%	
	Other	21.76%	
Ethnicity	Hispanic	14.71%	
BMI (Kg/m^2^)		29.1	6.6
Mental health	Days with poor mental health (past month)	3.21	7.30
Anxiety	≥15 days (past month)	12.61%	
Depression	≥15 days (past month)	6.01%	
Exercise (in minutes)	Moderate/vigorous exercise	124	187
Education	College graduate	26.51%	
	Less than high school	21.30%	
	High school graduate	25.88%	
	Some college education	26.31%	
Income (per year)	>75,000	29.79%	
	<20,000	17.24%	
	20,000–25,000	7.65%	
	25,000–35,000	12.45%	
	35,000–45,000	10.40%	
	45,000–55,000	8.78%	
	55,000–65,000	7.03%	
	65,000–75,000	6.66%	
General heath	Excellent	16.77%	
	Very good	29.57%	
	Good	34.31%	
	Poor	15.80%	
	Very poor	3.55%	
Marital status	Married	63.04%	
	Widowed	9.43%	
	Divorced/separated	11.11%	
	Never married	12.29%	
	Living with partner	4.13%	
Insurance status	Insured	85.35%	
Caffeine use	Present	91.06%	
Smoker	Yes	11.03%	
Daytime sleepiness	Present	15.07%	
Sleep duration	Sleep duration (hrs)	6.91	1.36
Insomnia symptoms	Difficulty falling asleep (DFA, ≥5 nights/month)	16.05%	
	Difficulty maintaining sleep (AWAK, ≥5 nights/month)	18.15%	
	Nonrestorative sleep (NRS, ≥5 nights/month)	22.32%	
Alcohol quantity	Drinks/day (past 12 months)	1.25	2.28
Drinking frequency	Drinking days (past 12 months)	40.9	85.8
Heavy drinking status	Present (past 12 months)	9.77%	
Heavy drinking frequency	Heavy drinking days (past 12 months)	4.23	27.18

S.D.: standard deviation.

**Table 2:** Associations of daytime sleepiness with alcohol variables and sleep duration.

Predictor	Subgroup	Model 1		Model 2		Model 3	
		OR (95% CI)	*P *	OR (95% CI)	*P *	OR (95% CI)	*P *
Alcohol	Log drinks/day	0.72 (0.58–0.90)	0.0046	0.78 (0.60–1.01)	0.0557	0.74 (0.58–0.95)	0.0197
	Log drinking frequency	0.87 (0.81–0.93)	0.0001	0.91 (0.84–0.99)	0.0297	0.90 (0.83–0.98)	0.0129
	Heavy drinking status	0.68 (0.43–1.07)	0.0971	0.76 (0.48–1.21)	0.2471	0.71 (0.45–1.11)	0.1311
	Log heavy drinking frequency	0.87 (0.74–1.02)	0.0805	0.89 (0.75–1.06)	0.1891	0.88 (0.75–1.04)	0.1366
Sleep duration	Hours	0.71 (0.64–0.80)	<0.0001	0.75 (0.67–0.84)	<0.0001	0.75 (0.67–0.84)	<0.0001

OR: odds ratio, CI: confidence interval, *P*: *P* value, and mo: months.

Model 1: unadjusted model.

Model 2: adjusted for age, BMI, gender, race, marital status, education, income, depression, insurance, health status, anxiety, mental health, and exercise.

Model 3: model 2 + insomnia status.

**Table 3:** Daytime sleepiness and its association with an interaction of alcohol consumption variables on sleep duration.

Moderating variable	Model 1 (*P*)	Model 2 (*P*)	Model 3 (*P*)
Log drinks/day	0.131	0.627	0.626
Log drinking frequency	0.323	0.628	0.602
Heavy drinking status	0.059	0.286	0.289
Log heavy drinking frequency	**0.005**	**0.003**	**0.004**

*P*: *P* value.

Model 1: unadjusted model.

Model 2: adjusted for age, BMI, gender, race, marital status, education, income, depression, insurance, health status, anxiety, mental health, and exercise.

Model 3: model 2 + insomnia status.
